# Investigating the Sulfonated Chitosan/Polyvinylidene Fluoride-Based Proton Exchange Membrane with fSiO_2_ as Filler in Microbial Fuel Cells

**DOI:** 10.3390/membranes13090758

**Published:** 2023-08-25

**Authors:** Gowthami Palanisamy, Ajmal P. Muhammed, Sadhasivam Thangarasu, Tae Hwan Oh

**Affiliations:** Department of Chemical Engineering, Yeungnam University, Gyeongsan 8541, Republic of Korea; ajmalpm127@gmail.com (A.P.M.); sadhasivam.nano@gmail.com (S.T.)

**Keywords:** chitosan, polyvinylidene fluoride, proton exchange membrane, microbial fuel cell, proton conductivity, inorganic filler, mechanical stability, functionalized SiO_2_

## Abstract

Chitosan (CS), a promising potential biopolymer with exquisite biocompatibility, economic viability, hydrophilicity, and chemical modifications, has drawn interest as an alternative material for proton exchange membrane (PEM) fabrication. However, CS in its original form exhibited low proton conductivity and mechanical stability, restricting its usage in PEM development. In this work, chitosan was functionalized (sulfonic acid (-SO_3_H) groups)) to enhance proton conductivity. The sulfonated chitosan (sCS) was blended with polyvinylidene fluoride (PVDF) polymer, along with the incorporation of functionalized SiO_2_ (–OH groups), for fabricating chitosan-based composite proton exchange membranes to enhance microbial fuel cell (MFC) performances. The results show that adding functionalized inorganic fillers (fSiO_2_) into the membrane enhances the mechanical, thermal, and anti-biofouling behavior. From the results, the PVDF/sCS/fSiO_2_ composite membrane exhibited enhanced proton conductivity 1.0644 × 10^−2^ S cm^−1^ at room temperature and increased IEC and mechanical and chemical stability. Furthermore, this study presents a revolutionary way to generate environmentally friendly natural polymer-based membrane materials for developing PEM candidates for enhanced MFC performances in generating bioelectricity and wastewater treatment.

## 1. Introduction

The globe is now facing two significant challenges: (i) energy shortage-related difficulties and (ii) environmental pollution (air pollution kills over 7 million people annually) [1,2,3,4,5]. Energy consumption and demands are rising at an unsustainable rate due to the world’s expanding population. However, the production of energy from fossil fuels is insufficient to fulfill the projected fuel demand. The increased release of greenhouse gases during fossil fuels’ extraction and energy conversion process profoundly impacts the environment. It is thought to contribute to the warming of the earth’s atmosphere. Another way that the industrial revolution contributes to the accumulation of solid waste is by allowing people to meet their necessities. Moreover, most municipal solid waste is not successfully degraded and needs to be properly transported to land fields and water mediums [6]. Most solid wastes are decomposed in open conditions using conventional methods, significantly increasing emissions of greenhouse gases [7]. Recently, the waste-to-energy (W2E) idea has provided tremendous progress in energy production development via waste degradation [8,9].

Consequently, the microbial fuel cell (MFC) has been selected and is regarded as one of the best technologies for producing energy from waste biomass [10,11]. MFC produces bioenergy with zero net carbon dioxide emissions, falling under the renewable energy category [12]. Different kinds of MFC devices have been constructed to attain efficient performances based on the alignment, components, arrangements, and conditions, and have been coupled with other options [13,14,15,16,17,18]. The essential advantages of MFC are the simultaneous treatment of wastewater and the production of electricity in a single unit [19,20]. Various kinds of electrogenic microorganism aids in degrading the wastes (organic matter), such as oxidizing organic compounds from the water and generating electricity in MFCs [7,21,22]. During the substrate (natural or synthetic wastewater) degradation, the electron and proton are released by the oxidation process on the anode side [19,23]. MFC’s next steps are similar to the proton-exchange membrane fuel cell. The electron is collected and transferred to an electric circuit via the electrode in an anode chamber. The overall reaction process is completed in the cathode by an oxygen reduction reaction with the oxygen, proton, and electron, where the electron and proton are generated in the anode and transferred to the cathode via an external electric circuit and proton selective membranes, respectively. Using acetic acid, the possible MFC reaction process is as follows (Anode reaction: CH_3_COOH + 2H_2_O → 2CO_2_ + 8H^+^ + 8e^−^; Cathode reaction: 8H^+^ + 8e^−^ + 2O_2_ → 4H_2_O; Overall reaction: CH_3_COOH + 2O_2_ → 2H_2_O + 2CO_2_) [17].

The separator/membrane is one of the most noticeable components in MFC, and the membrane also determines MFC performance [24,25,26,27]. In MFC, the prominent role of the membrane is to conduct the ion transport from the anode to the cathode to complete the reaction process, prohibiting the substrate cross-over and contact between the anode and cathode electrodes. Moreover, the membrane materials have effectively influenced the system’s internal resistance [28]. The following characteristic properties are most considered for favorable membranes: less-expensive, high ion selectivity, high mechanical/chemical/thermal stability, escaping from fouling-related issues, and biocompatibility. In dual-chambered MFC, the cation exchange membrane (CEM) effectually showed considerable benefits because of its proton conduction behavior, which is most favorable for obtaining higher performances. However, in most cases, any of the following demerits are associated with the CEM, such as high production cost, substrate/oxygen diffusion, and biofouling [29,30]. Perfluoro sulfonic acid polymer (PFSA) membranes such as Nafion (DuPont), Hyflon (Solvay-Solexis), Flemion (Asahi Glass), and 3M were found to be an efficient membrane material for MFC systems because of their excellent intrinsic properties towards proton transportation and stability [28]. However, PFSA-based membrane exhibits high production cost, substrate loss, and a high oxygen transfer rate, which limits their benefits from the commercial viewpoint [30]. Numerous attempts have been made to use different types of natural and synthetic polymer materials as a membrane option for MFC applications to address these difficulties.

Thermoplastic properties of the fluoropolymer category of polyvinylidene fluoride (PVDF) have been considered for preparing the membrane for various applications such as fuel cells [31], batteries [32], water treatment [33,34], tissue engineering [35], etc., because of PVDF’s low production cost and its intrinsic properties such as efficient chemical resistance, thermal stability, inherent resistance, biocompatibility, electrical insulation, and mechanical stability [36,37,38]. In this connection, the efforts on PVDF-based membranes were made with different concepts using different materials and processes such as blend, composite, cross-linked, porous, and reinforced membranes for MFC [36,39,40,41]. The PVDF with a synthetic fluoropolymer (such as PFSA) and hydrocarbon polymers has been well established [38,42,43,44]. The combination of PVDF and biopolymers materials has received some attention recently for improving the characteristics of PVDF-based membranes. Biopolymers probably offer the essential properties of ion exchange capacity and ionic conductivity for PVDF-based membranes. Moreover, biopolymers have considerable benefits such as natural abundance, low production/processing cost, biocompatibility, and a considerable amount of water molecules holding behavior [30,45,46,47]. In this view, chitosan (CS) has been effectively considered a membrane for MFC applications because of affirmative behavior, as mentioned above. However, the direct utilization of CS as a membrane is associated with significant drawbacks because of their higher amount of swelling nature, which severely affects the mechanical stability and selectivity of membranes and lesser proton conductivity [45]. Thus, different efforts, specifically surface modification of CS, blend, cross-linking, and composites, were developed to overcome the CS-related issues [48,49,50,51]. Thus, the properties of PVDF can be further altered by including the desired amount and functional properties of CS in the PVDF membrane matrix (PVDF-CS). Moreover, incorporating inorganic additives as a filler in the polymer matrix can further influence membrane properties for MFC performances [52,53,54,55]. In this regard, various additives have been employed for membranes for MFC. However, very few studies on combining synthetic polymer–biopolymer–inorganic additives (SP-BP-IA) have been investigated for the MFC system. In this viewpoint, we established the new composite formation of SP-BP-IA. The modification of CS by introducing a sulfonic acid functional group (-SO_3_H) (sCS) effectively influences the proton transportation behavior and is confirmed for various applications. Abbas Shirdast et al. revealed the impact of sulfonation in CS, where the proton conductivity of CS and CS/sCS membranes are 1.30 ± 0.06 and 2.20 ± 0.11 mS cm^−1^, respectively [56]. Moreover, the incorporation of SiO_2_ as a nanofiller for membranes has been well studied for various fuel cell applications, which can effectively influence the MFC performances by controlling the substrate crossover, enhancing the mechanical stability, chemical stability, and proton transport behavior, and controlling the membrane’s dimensional stability.

In this study, we established a new kind of membrane in the concept of synthetic polymer–biopolymer–inorganic additives for proposing the possibilities for MFC. For developing the PVDF-based hybrid membrane, we modified CS by functionalizing the -SO_3_H as sulfonated CS (sCS) and the SiO_2_ by introducing the –OH group on the surface. The hybrid PVDF/sCS/SiO_2_ has been developed through a solution casting technique. The efficient membrane was identified by two steps: (i) changing the ratio between PVDF and as-developed sCS, and (ii) introducing different wt.% of –OH functionalized SiO_2_ in the PVDF/sCS. Structural and microstructural analyses were used to identify the possibilities of materials and membranes modification. An efficient membrane was identified by measuring different properties such as thermal, mechanical, and hydrophilic characteristics, ion-exchange capacity, and proton conductivity. Thus, the PVDF/sCS/SiO_2_ composite membrane has been optimized in the present investigation and proposed for MFC applications. 

## 2. Materials and Methods

### 2.1. Synthesis of Sulfonated Chitosan (sCS)

A series of steps were followed to synthesize sulfonated chitosan (sCS) [57]. Initially, 1 g of chitosan (CS) was dissolved in a 100 mL solution of 1.0 wt.% aqueous acetic acid at 25 °C. The CS solution obtained was continuously stirred under a nitrogen (N_2_) atmosphere for a period of 30 min. Subsequently, 1,3-Propane sultone (PS), weighing 2.0 g, was added drop by drop to the CS solution while maintaining the N_2_ environment. The resulting mixture was stirred at 40 °C for 5 h. Once the reaction was complete, the product was slowly introduced into a cold solution of acetone. This caused the formation of a white flocculent material, which precipitated out of the solution. To ensure the removal of any residual PS, the flocculent material was then subjected to filtration and washed with methanol 4–6 times. Following this, the resulting product was dried thoroughly and subsequently labeled as sCS.

### 2.2. Preparation of Functionalized SiO_2_

Functionalized SiO_2_ (fSiO_2_) was prepared by incorporating hydroxyl (-OH) functional groups onto the surface of SiO_2_. Initially, the SiO_2_ sample underwent a heat treatment at 500 °C for 48 h in a furnace to eliminate any impurities. Following this, a specific quantity of SiO_2_ was combined with 30 mL of 1 M ethanol solution. The mixture was then subjected to ultrasonication for a duration of 30 min to yield SiO_2_-OH.

### 2.3. Preparation of Composite Membrane

All membranes, including the pristine PVDF, PVDF/SiO_2_, PVDF/fSiO_2_, and PVDF/sCS/fSiO_2_ membranes, were fabricated using the solvent casting method. The process began with the preparation of a 10 wt.% PVDF solution by dissolving PVDF in DMAc, which was then stirred for 24 h at 60 °C to achieve homogeneity. The homogenous PVDF solution was cast onto a clean, dry glass plate using a 300 µm casting knife, and the cast solution was dried at 60 °C for 24 h to obtain the pristine PVDF membrane.

To fabricate the inorganic composite membranes, namely PVDF/SiO_2_ and PVDF/fSiO_2_, desired proportions of SiO_2_ (3 wt.% relatives to PVDF) and fSiO_2_ (1 wt.%, 2 wt.%, or 3 wt.% relatives to PVDF) were dispersed into separate vials containing DMAc through ultrasonication. Subsequently, the desired amount of PVDF was added to each inorganic suspension, and the membranes were prepared using the same procedure as the pristine PVDF membrane. The resulting membranes were labeled as PVDF/SiO_2_, PVDF/fSiO_2_ 1%, PVDF/fSiO_2_ 2%, and PVDF/fSiO_2_ 3%. For the fabrication of the PVDF/sCS/fSiO_2_ membrane, the inorganic suspension of fSiO_2_ in DMAc was prepared as described earlier. The desired amount of sCS in PVDF solution at the ratio of 1:9 was prepared and added to the solution, followed by an additional 24 h of stirring. Membranes were prepared using the procedure as mentioned earlier. The resulting membranes were designated as PVDF/sCS/fSiO_2_ membranes.

### 2.4. Characterization

Fourier transform infrared spectroscopy (FTIR) analysis was conducted using a Perkin Elmer FT–IR spectrometer (Spectrum 100, Shelton, CT, USA). The FTIR spectra were recorded over a wavenumber range of 4000 to 600 cm^−1^. X-ray diffraction (XRD) patterns were obtained using an X-ray diffractometer (model: Xpert Pro, Malvern, UK). The measurements were performed in a diffraction angle (2θ) range of 5° to 50° with a scan step size of 0.02°. Cu Kα radiation with a wavelength (λ) of 1.54 Å was employed for the analysis. The surface microstructural characteristics of the samples were observed using a scanning electron microscope (SEM, Hitachi S-4800, Ibaraki, Japan). The analysis was performed at a 10 kV acceleration voltage. To prevent any potential electrostatic charging during the examination, a thin layer of platinum was deposited on the samples using an ion-sputter physical vapor deposition machine (Hitachi E-1030, Ibaraki, Japan). An atomic force microscope (AFM) was performed to study the surface roughness and topography of the membranes in a non-contact mode (n = 5).

The thermal stability of the membranes was assessed using thermogravimetric analysis (TGA, SDT Q600, TA Instruments, New Castle, DE, USA) with a heating rate of 10 °C/min. Samples weighing approximately 5 mg were heated from 30 °C to 800 °C in a nitrogen atmosphere. The mechanical properties (tensile strength (TS) and elongation at break (EAB)) of the composite membranes were assessed by ASTM standard method D882, utilizing a universal testing machine (3345, INSTRON, Norwood, MA, USA). Precise strips measuring 60 mm × 10 mm were obtained from the membrane samples using a sharp cutting knife. The measurements were conducted with an initial grip separation of 30 mm and a crosshead speed of 5 mm/min.

To evaluate the surface wettability of the membrane samples, the sessile drop method was employed with the assistance of a contact angle analyzer (OCA 20 analyzer, dataphysics, Filderstadt, Germany). A small droplet of DI water (10 μL) was placed on 1 cm × 1 cm membrane specimens, and the contact angle of the water droplet on the membrane surface was measured. The membrane samples were cut into squares measuring 3 cm × 3 cm and subjected to drying in an air oven at 50 °C until a constant dry weight was achieved. Subsequently, the dry weight (*W_dry_*) and dry area (*A_dry_*) of each sample were recorded. Following this, the samples were immersed in deionized (DI) water and allowed to soak thoroughly for a duration of 24 h. After the soaking period, the samples were carefully taken out, excess surface water was gently removed using filter paper, and the wet weight (*W_wet_*) and the wet area (*A_wet_*) were measured.

The water uptake (WU) of the membrane was calculated using the formula:

$$\mathrm{WU}\;(\%)=\frac{W_{wet}-W_{dry}}{W_{dry}}\times 100$$

The membrane swelling ratio (SR) was calculated using the formula:

$$\mathrm{SR}\;(\%)=\frac{A_{wet}-A_{dry}}{A_{dry}}\times 100$$

To analyze the ion exchange capacity (IEC) of a membrane, the pre-dried membrane samples were cut into small pieces of 4 cm × 4 cm dimension, and their dry weight was noted as *W_dry_*. These samples were then immersed in 100 mL of 1 M NaCl solution for 24 h, ensuring they are fully submerged. This step opens up the ion exchange sites, replacing H^+^ ions with Na^+^ ions. After removal from the solution, the solution containing the released protons was titrated using phenolphthalein as an indicator against a 0.01 M NaOH solution. The volume of NaOH solution used during the titration was recorded as *V_NaOH_*, and the concentration of the NaOH solution was noted as *C_NaOH_*. The ion exchange capacity (IEC) was calculated using the formula:

$$\mathrm{IEC}=\frac{V_{NaOH}\times C{\;}_{NaOH}}{W_{dry}}$$

The membrane’s proton conductivity (σ) was determined using electrochemical impedance spectroscopy with the aid of a Corrtest instrument. An electrochemical Teflon cell was set up, utilizing a membrane with dimensions of 6 cm × 1 cm, sandwiched between two stainless steel electrodes. Deionized water served as the electrolyte solution. The impedance response was measured across a frequency range from 100 MHz to 1 MHz. The proton conductivity (σ) was measured using the given equation:

$$\mathrm{Proton}\;\mathrm{conductivity}\;(S/\mathrm{cm})=\frac{D}{R\times A}$$

where *D* represents the distance between the two electrodes in centimeters, *R* denotes the resistance calculated from the Nyquist impedance plot in ohms, and *A* represents the electrode area.

## 3. Results and Discussion

### 3.1. Characterization of Sulfonated Chitosan (sCS)

**Figure 1:**
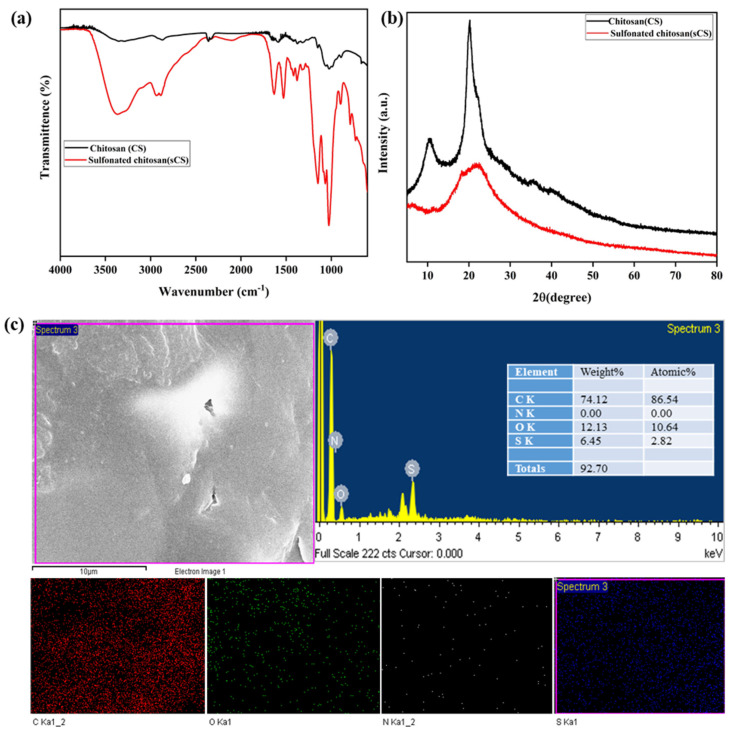
(**a**) FTIR spectra, (**b**) XRD, and (**c**) FESEM−EDAX analysis of chitosan (CS) and sulfonated chitosan (sCS).

FTIR spectra of chitosan and sulfonated chitosan (sCS) are shown in Figure 1a. It exhibited evidence for successful chitosan sulfonation and attachment of hydroxyl groups into SiO_2_. The FTIR spectrum of chitosan (CS) revealed the existence of characteristic broad peaks at 3300–3400 cm^−1^, which corresponds to the stretching vibration of hydrophilic groups such as –OH and –NH_2_, as well as strong intermolecular hydrogen bonding. The characteristic peak at 2868 cm^−1^ is related to the –CH_2_ stretching vibration [58]. The stretching vibration of the –NH_2_ group was observed in the range of 1594 cm^−1^. The peak at 1377 cm^−1^ shows the bending vibrations of the methylene group. Additionally, the C-O stretching vibrations in glycosidic bands were observed in the 1115 cm^−1^ and 1075 cm^−1^ range [59]. The skeletal vibration involving the C-O stretching was observed at 1025 cm^−1^ [60]. It has been observed that the FTIR spectrum of sCS showed some variable features along with characteristic transmittance peaks of pristine CS. The distinct chitosan peak at 3357 cm^−1^ becomes narrower, which explains the inter- and intramolecular hydrogen bond weakening of CS [61]. The C-O stretching vibration of the amide group and C-N-C bending vibration in sCS were attributed to the strong peaks at 1629 cm^−1^ and 1531 cm^−1^ [62]. The intense absorption peaks at 1025 cm^−1^ observed the O-S-O stretching vibration. The asymmetrical stretching vibration absorption and symmetrical stretching vibration absorption of sulfate were marked by the peak at 1382 cm ^−1^ [63]. The band around 791 cm^−1^ and 894 cm^−1^ corresponds to C-O-S stretching vibrations.

The X-ray diffraction patterns of chitosan (CS) and sulfonated chitosan (sCS) are shown in Figure 1b. It has been observed from the Figure that chitosan exhibited two reflection peaks at 2θ = 10.45° and 2θ = 20°, which corresponds to crystal forms I and crystal forms II, respectively [63]. For sulfonated chitosan, the crystalline peak intensity at 2θ = 20° has decreased with the disappearance of the peak at 2θ = 10.45°. This shows that the sulfonation process reduces the hydrogen bond formation in sulfonated chitosan owing to incompetent packing and weak sites of CS chains [64,65,66]. As a result, the crystalline phase of chitosan crystal form I and form II has decreased with a more significant portion of the amorphous phase in sulfonated chitosan [61]. Figure 1c illustrates the results obtained from the EDAX analysis of sulfonated chitosan (sCS) during FESEM, confirming the presence of sulfur (S) and C, N, and O elements. The sulfonation of chitosan has been confirmed from elemental dot mapping, which showed the homogenous distribution of C, N, O, and S elements.

### 3.2. Characterization of Composite Membranes

#### 3.2.1. Structural Characterization

**Figure 2:**
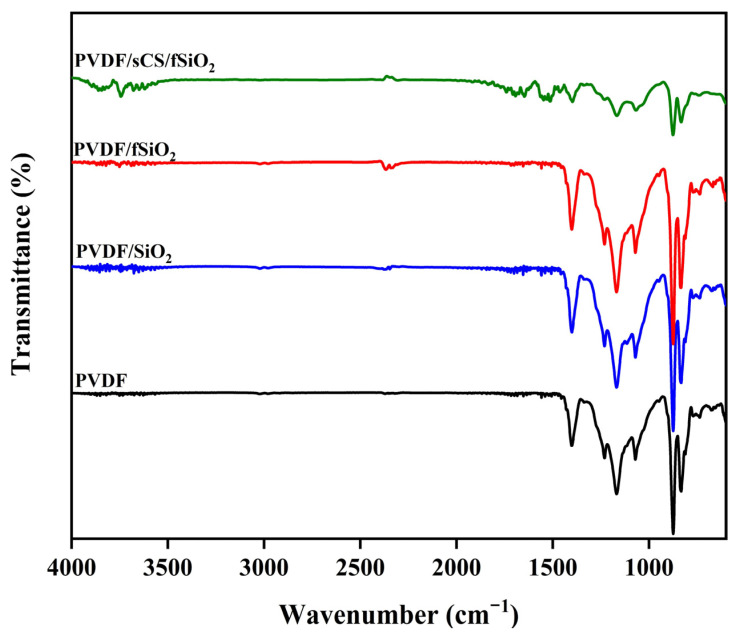
FTIR spectra of pristine PVDF, PVDF/SiO_2_, PVDF/fSiO_2_ and PVDF/sCS/fSiO_2_ composite membranes.

FTIR spectra of PVDF, PVDF/SiO_2_, PVDF/fSiO_2_, and PVDF/sCS/fSiO_2_ composite membranes are shown in Figure 2. For the FTIR spectra of pure PVDF membrane, the absorption peak at 1400 cm^−1^ indicated the –CH_2_ wagging vibration. The peak at 1165 cm^−1^ showed the C-C band in PVDF [67]. The 1229 cm^−1^ and 823 cm^−1^ peaks revealed the –CF_2_ stretching vibration [68]. The C-C-C asymmetrical stretching vibration of the PVDF membrane was attributed to a peak at 874 cm^−1^ [68]. The characteristic absorption peaks of pure PVDF membranes were observed to be conserved in the spectrum of composite membranes. The peak at 1110 cm^−1^ in the PVDF/SiO_2_ composite membranes indicated the Si-O-Si asymmetrical stretching vibrations. The PVDF/sCS/fSiO_2_ composite membranes exhibited the –OH stretching vibration around 3700 cm^−1^. The stretching vibration of the –NH_2_ group of chitosan was observed at 1636 cm^−1^ along with –CH_2_ deformation vibration at 1461 cm^−1^, and the peak at 1396 cm^−1^ indicated the asymmetric and symmetric stretching vibration absorption of sulfate [63].

**Figure 3:**
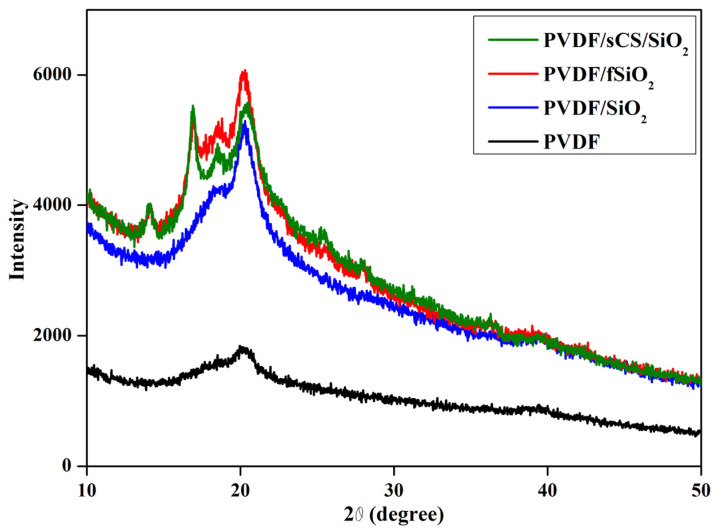
XRD pattern of pristine PVDF, PVDF/SiO_2_, PVDF/fSiO_2_, and PVDF/sCS/fSiO_2_ composite membranes.

Figure 3 illustrates the XRD pattern of pristine PVDF, PVDF/SiO_2_, PVDF/fSiO_2,_ and PVDF/sCS/fSiO_2_ composite membranes. For pristine PVDF membrane, the diffraction pattern of 2θ = 20° indicated the β-phase of PVDF, and a small diffraction pattern at 2θ = 39° indicated the α-phase was observed. The composite membranes exhibited a similar diffraction pattern to pristine PVDF membranes. From there, it has been observed that base PVDF polymer has been unaffected by the addition of inorganic SiO_2_ and sulfonated CS. The incorporation of SiO_2_ enhances the amorphous behavior of the composite membrane with increased membrane flexibility [44]. The hydrophilic properties of the membrane have been reduced by membrane crystallinity that alters the WU values, affecting the membrane proton conductivity. Hence, a decrease in membrane crystallinity enhances the proton conductivity of the membrane [69]. The diffraction pattern overlaps with the base polymer pattern for PVDF/sCS/fSiO_2_ composite membrane. The low-intensity peaks observed in composite membranes have resulted from residues obtained during membrane formation. The intermolecular interaction between the polymers in the composite membrane destructed the hydrogen bonding network and reduced the crystallinity. Thus, this enhanced the free mobility of polymer chains, attributed to the increased membrane proton conductivity [70].

#### 3.2.2. Morphological Analysis

**Figure 4:**
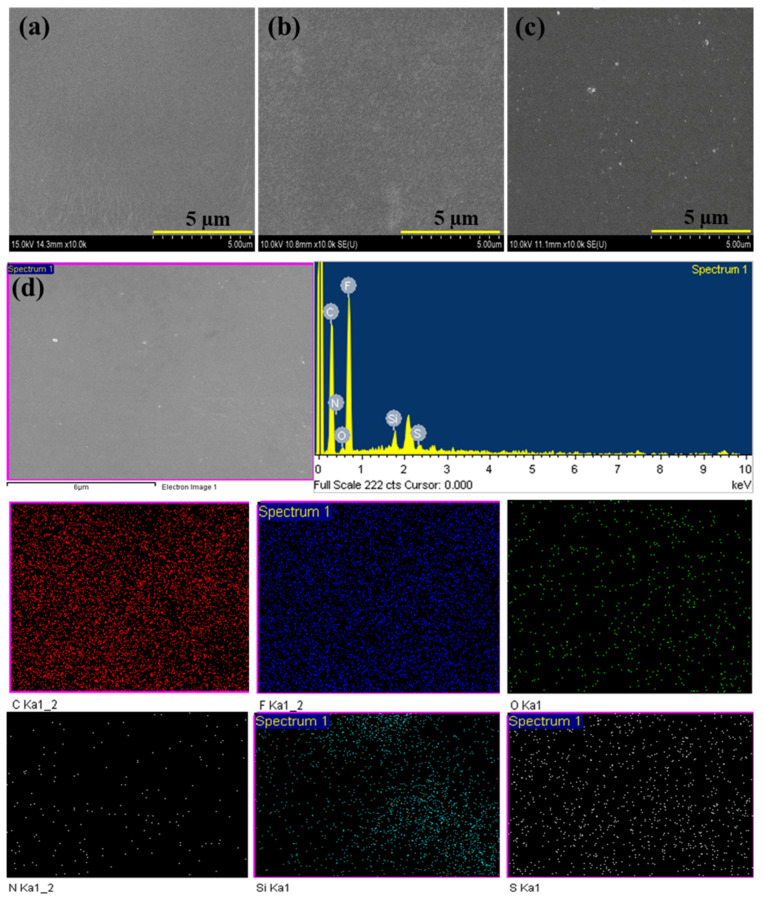
FESEM images of (**a**) pristine PVDF, (**b**) PVDF/SiO_2,_ and (**c**) PVDF/sCS/fSiO_2_ membranes; and (**d**) FESEM−EDX images of PVDF/sCS/fSiO_2_ membrane.

The surface morphology of pristine PVDF, PVDF/SiO_2,_ and PVDF/sCS/fSiO_2_ composite membranes are shown in Figure 4a–c. It has been identified that the pristine PVDF membrane exhibited smoothness and flatness without any pinholes (Figure 4a). From Figure 4b, the morphology of the PVDF/SiO_2_ composite membrane exhibited slight roughness due to the incorporation of inorganic SiO_2_-OH fillers. According to Figure 4c, the surface morphology of the PVDF/sCS/fSiO_2_ composite membrane exhibited some white agglomerates on the top surface. This may be due to tiny undissolved polymers (PVDF or sCS) or the patchy dispersion of inorganic fillers. Here, the membrane phase separation was not observed, indicating a homogenous surface without irregular structures. The EDAX analysis of the PVDF/sCS/fSiO_2_ composite membrane (Figure 4d) reveals the uniform dispersion of C, F, O, N, Si, and S elements. The presence of Si and S elements in the composite membrane is derived from fSiO_2_ (SiO_2_-OH) fillers and sCS, confirming all elements’ homogenous dispersion.

**Figure 5:**
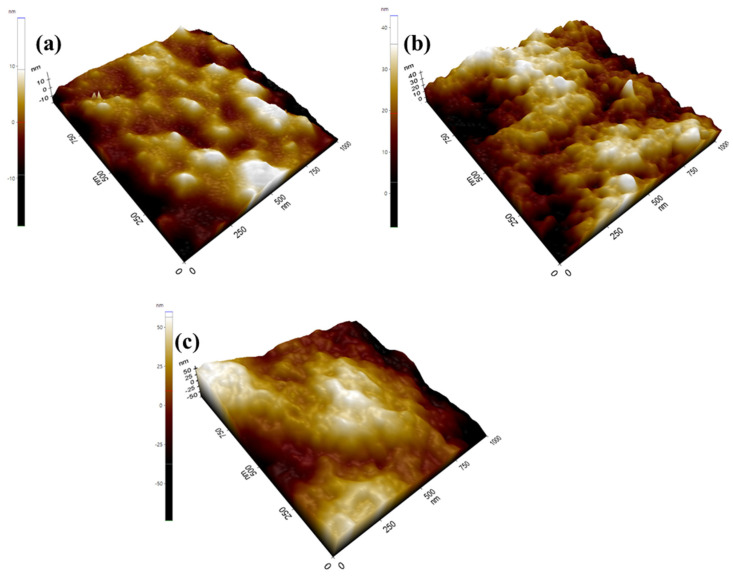
AFM images of (**a**) pristine PVDF, (**b**) PVDF/SiO_2_, and (**c**) PVDF/sCS/fSiO_2_ membranes.

The phase morphology, such as the surface roughness of the pristine PVDF, PVDF/SiO_2,_ and PVDF/sCS/fSiO_2_ composite membranes, was analyzed through AFM and shown in Figure 5. The composite membranes’ hydrophilic/hydrophobic domain morphologies are denoted by light and dark regions, as depicted by AFM micrographs. From this analysis, the performance of MFC has been analyzed. From the figure, it has been observed that pristine PVDF membrane displays smooth surfaces, while the incorporation of inorganic filler enhances the membrane roughness. In the same way, the membrane roughness increased with the presence of sulfonated chitosan (sCS) in the PVDF/sCS/fSiO_2_ composite membrane. Additionally, enhancement in membrane roughness improves the membrane hydrophilicity, and thus membrane proton conductivity increases [71]. It is well known that an increase in membrane roughness enhances the surface area, leading to the development of thin biofilms on the membrane surface. As a result, the amount of oxygen transferred from the cathodic chamber to the anodic chamber has been reduced. Thus, the anodic chamber’s enrichment to the aerobic environment has been enhanced, ultimately increasing the MFC efficacy [72].

#### 3.2.3. Membrane Hydrophilic Properties

**Figure 6:**
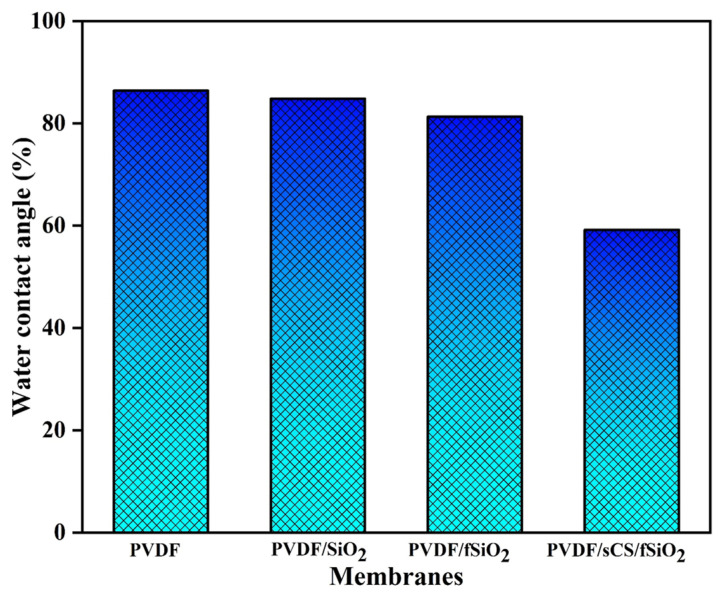
Water contact angles of pristine PVDF, PVDF/SiO_2_, PVDF/fSiO_2_ and PVDF/sCS/fSiO_2_ composite membranes.

The water contact angle measurements of the pristine PVDF, PVDF/SiO_2_, PVDF/fSiO_2,_ and PVDF/sCS/fSiO_2_ composite membranes exhibited the hydrophilic membrane properties and were shown in Figure 6. The contact angle measurements show the strongest correlation between the membrane hydrophilicity and membrane roughness. The PEM in MFC should possess good anti-fouling properties [39]. It has been identified that membranes with increased hydrophilic characteristics exhibited enhanced anti-fouling properties. In the figure, the pristine PVDF membrane showed the hydrophobic characteristic observed from their contact angle values of 86.4° with the lowest R_a_ value of 3.634 nm. Incorporating the inorganic SiO_2_ fillers in the composite membranes decreases the water contact angle values and increases the membrane hydrophilicity. Meanwhile, it was also observed that PVDF/sCS/fSiO_2_ composite membrane exhibited the lowest contact angle value (59.2°) and higher R_a_ (19.169 nm). This resulted in the enhancement of the membrane’s hydrophilic properties. This hydrophilic increment was attributed to the incorporation of –SO_3_H groups in sCS and also the hydrophilic nature of CS in the composite membrane. In other words, it has been explained that the presence of amino, –OH, and sulfonic groups and the exceptional hydration behavior of the PVDF/sCS/fSiO_2_ composite membrane resulted in enhanced hydrophilic properties [73]. These results indicate that hydrophilic membrane properties are greatly influenced by membrane topological behavior and the presence of –SO_3_H groups in the PVDF/sCS/fSiO_2_ composite membrane.

#### 3.2.4. Thermal and Mechanical Stability

**Figure 7:**
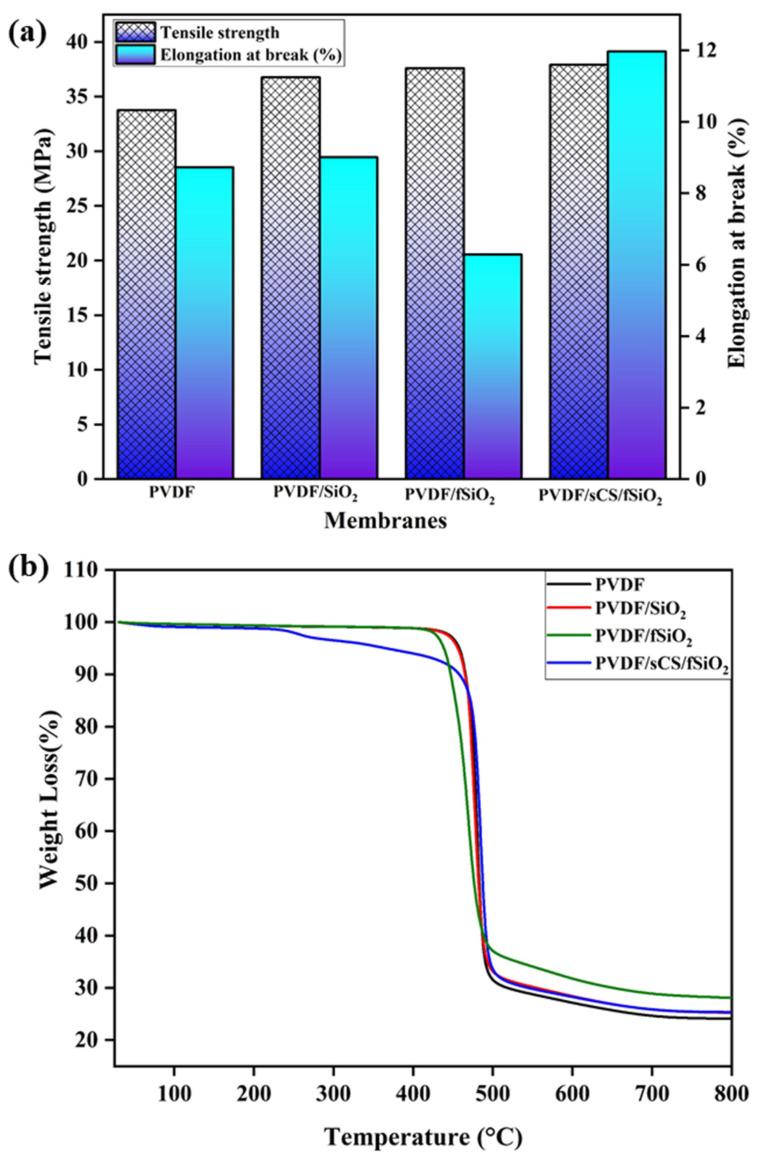
(**a**) Tensile strength, elongation at the break and (**b**) TGA analysis of the pristine PVDF, PVDF/SiO_2_, PVDF/fSiO_2_, and PVDF/sCS/fSiO_2_ composite membranes.

The thermal stability of the membranes (pristine PVDF, PVDF/SiO_2_, PVDF/fSiO_2_, and PVDF/sCS/fSiO_2_ composite membranes) were analyzed through thermogravimetric analysis (TGA) techniques and exhibited in Figure 7b. In pristine PVDF membrane, little weight loss has been observed at ~200 °C that was attributed to the moisture loss along with polymeric fluoride (C-F) chain degradation [74]. Following increase in temperature, about ∼65% weight loss was recorded at 500 °C, due to the breakdown of the PVDF polymer backbone chain. Moreover, incorporating SiO_2_ fillers in the membrane also exhibited a similar trend. A slight increase in thermal stability by the incorporation of inorganic SiO_2_ fillers was observed. Furthermore, three-step weight loss has been observed in the PVDF/sCS/fSiO_2_ composite membrane TGA curve. Initial weight loss around 25–220 °C was observed, owing to the evaporation of physically absorbed water, volatilization, and sublimation of small molecules. Around 220–500 °C, the second weight loss was identified due to the dissociation of functional groups, such as degradation of amino groups and decomposition of SO_3_H and linkage between CS along with the degradation of inorganic SiO_2_ filler and CS polymer backbone [73]. The final weight loss (above 500 °C) was apparently related to the deterioration of the PVDF polymer backbone. From these results, it has been observed that the presence of strong co-ordination bonds between the polymers and inorganic fillers by sCS enhances the compo-site membrane matrix stability and increases the membrane thermal stability.

The mechanical stability of the membrane was measured in terms of tensile strength (TS) and elongation at break (EAB). The membranes in MFC should sustain high hydrostatic pressure. Thus, the membranes should possess high mechanical stability. In Figure 7a, the pristine PVDF membrane exhibited a tensile strength value of 33.76 MPa. The incorporation of inorganic SiO_2_ filler into the PVDF enhanced the tensile strength of the composite membranes (36.77 MPa, 37.61 MPa, and 37.91 MPa for PVDF/SiO_2_, PVDF/fSiO_2_, and PVDF/sCS/fSiO_2_ composite membranes, respectively. This increment was attributed by the formation of a strong interaction between the inorganic filler and polymer matrix. Here, SiO_2_ may act as a reinforcing material in the membrane matrix. This enhances the membrane’s mechanical stability in terms of its tensile strength values [75]. Moreover, the inorganic SiO_2_ and functionalized SiO_2_ reduce the free movement of the polymer chain during stress environment by forming an interconnection between the polymer and inorganic SiO_2_ filler. Furthermore, the PVDF/sCS/fSiO_2_ composite membrane exhibited higher EAB values (11.97%) than all other membranes. The enhancement in EAB values was observed from the presence of hydrophilic functional groups (-SO_3_H groups) in sCS, which assisted in the formation of hydrogen bond network and electrostatic interactions. In addition to this, there was formation of the cross-linking network in the PVDF/sCS/fSiO_2_ composite membrane that reduces the unfolded polymer chain length and enhances the chain elongation [76]. Thus, the PVDF/sCS/fSiO_2_ composite membrane exhibited the increased mechanical stability values for MFC performances.

#### 3.2.5. Water Uptake, Swelling Ratio, and Ion-Exchange Capacity

**Figure 8:**
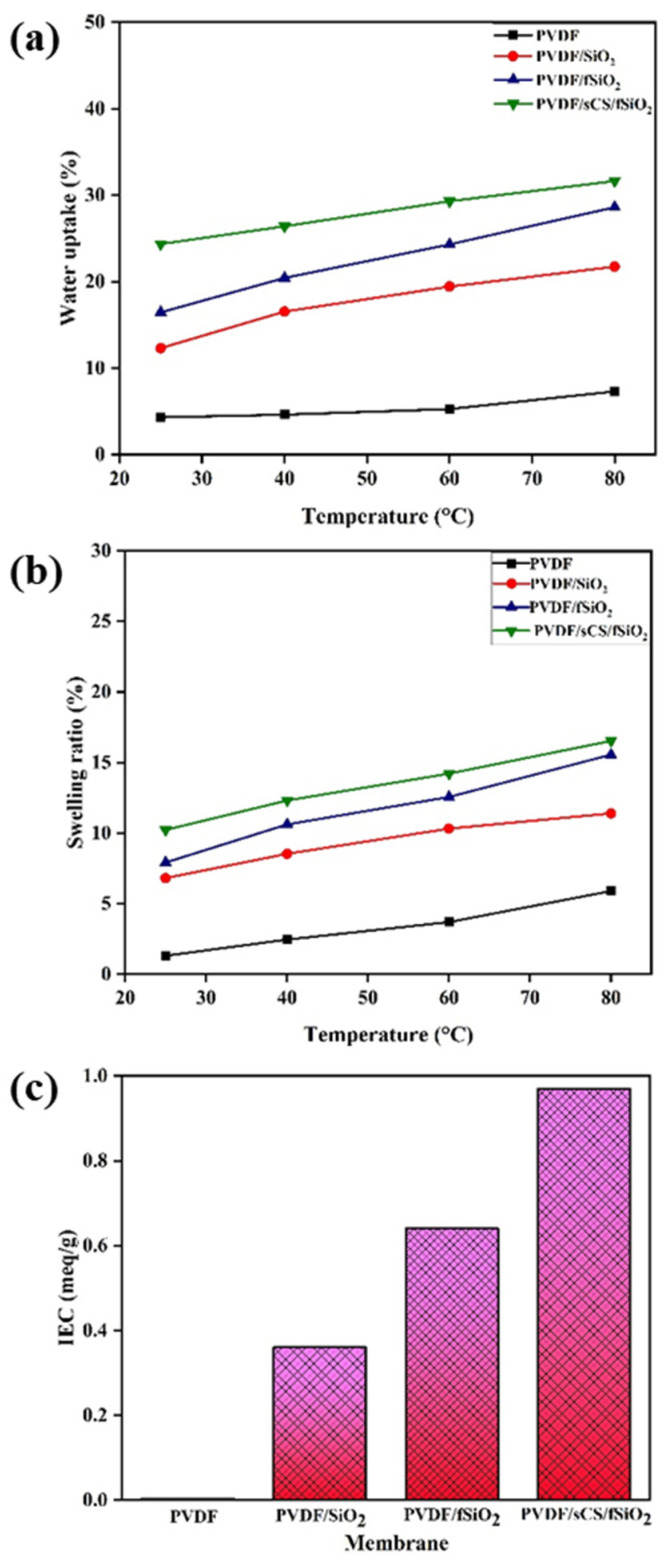
(**a**) Water uptake, (**b**) swelling ratio, and (**c**) ion exchange capacity of the pristine PVDF, PVDF/SiO_2_, PVDF/fSiO_2_, and PVDF/sCS/fSiO_2_ composite membranes.

The proton exchange membrane’s (PEM) water uptake values are regarded as a crucial membrane characteristic due to their effects on proton conductivity and mechanical stability. Increased water uptake values resulted in high proton conductivity values, which also causes undesirable effects on dimensional swelling, resulting in membrane mechanical deterioration. The rise in temperature enhances the polymer chain mobility resulted in the formation of additional room for water absorption [77]. Thus, an increase in temperature increases the membrane water uptake values. Figure 8a–c illustrates the water uptake, swelling ratio, and ion-exchange capacity values of the pristine PVDF, PVDF/SiO_2_, PVDF/fSiO_2,_ and PVDF/sCS/fSiO_2_ composite membranes. The pristine PVDF membrane exhibited very low WU properties due to its hydrophobic properties. The introduction of inorganic SiO_2_ fillers into the polymer matrix enhances its WU property due to the hygroscopic property of SiO_2_. This has also been supported by the presence of increased Lewis acid sites (responsible for attracting water molecules) in SiO_2_ [78]. Furthermore, during the functionalization of SiO_2_ fillers, many –OH groups are present, which offers more locations for forming hydrogen bonds between the polymer and water [79]. Moreover, the presence of sCS in the composite membrane enhances the WU values of the PVDF/sCS/fSiO_2_ composite membrane (25.74%). The presence of hydrophilic –OH, –NH_2_, and –SO_3_H groups in sCS enhances the membrane WU properties. Moreover, the hydrophilic groups in the composite membrane generated passages that was occupied by water molecules, thus enhancing the water uptake values [80]. Likewise, membranes with high water uptake generally possess high swelling ratio values, as shown in Figure 8b. Furthermore, the ion exchange capacity (IEC) of the polymeric membrane is also an essential property for membrane performance. It acts as an indicator of the existence of acidic groups, which are accountable for the conduction of protons, and it offers an accurate estimation of the proton conduction. IEC elucidates the information on the ionic charge responsible for the OH- exchange as measured by the number of functional group equivalents in the membrane per unit of dry weight [81]. However, a high IEC value will boost membrane water retention and proton conductivity while posing the risk of dimensional swelling [44]. Figure 8c shows the IEC values of pristine PVDF, PVDF/SiO_2_, PVDF/fSiO_2,_ and PVDF/sCS/fSiO_2_ composite membranes. The results show that PVDF/sCS/fSiO_2_ composite membranes exhibited higher IEC values (0.97 meq/g) due to the presence fixed charge sites such as sulfonic groups (–SO_3_H groups) of sCS that influences the IEC by enhance protons movement through hopping mechanisms [82].

#### 3.2.6. Proton Conductivity

**Figure 9:**
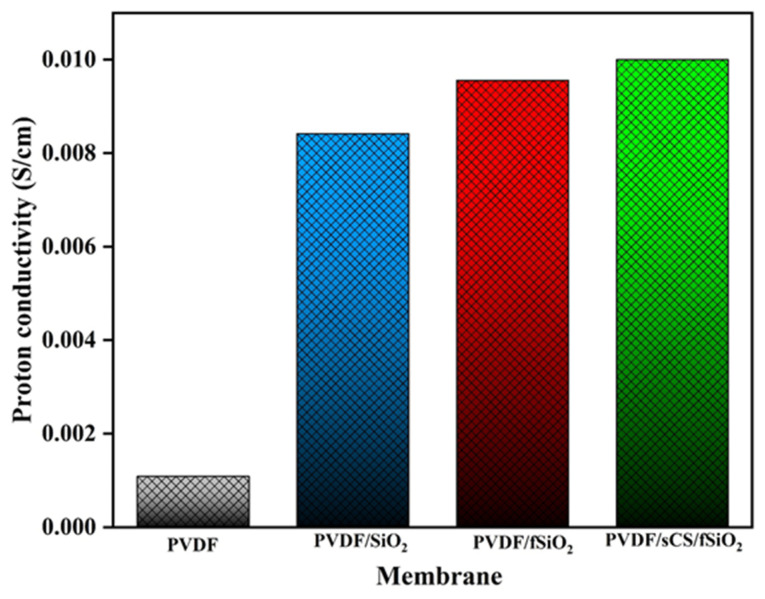
Proton conductivity of the pristine PVDF, PVDF/SiO_2_, PVDF/fSiO_2_ and PVDF/sCS/fSiO_2_ composite membranes.

Membrane proton conductivity plays a significant role in fuel cell performance in terms of power efficiency, including operational voltage and current output. Generally, proton conductivity mechanisms in the membrane rely on two pathways, namely (i) the vehicular mechanism involving the proton transport channels such as hydronium ions (namely H_3_O^+^, H_5_O_2_^+^, and H_9_O_4_^+^) in the transferring protons and (ii) the Grotthuss mechanism in which the proton transport occurs through conducting carrier (–SO_3_H groups) by hydrogen bonds [44]. For both these mechanisms, water availability in the membrane is vital. Here, free water molecules in the membrane are involved in the vehicular mechanism, while bound water is involved in the Grotthus mechanism. Hence, increased membrane WU enhances the proton conductivity of the membrane [70]. The proton conductivity measurements for the pristine PVDF, PVDF/SiO_2_, PVDF/fSiO_2,_ and PVDF/sCS/fSiO_2_ composite membranes were identified through Nyquist plots and exhibited in Figure 9. Incorporating inorganic SiO_2_ fillers into the polymer enhances the composite membrane’s proton conductivity through its hygroscopic properties [77]. The proton conductivity values of PVDF/SiO_2_, PVDF/f SiO_2_, and PVDF/sCS/fSiO_2_ composite membranes were found to be 8.418 × 10^−3^ S/cm, 9.56 × 10^−3^ S/cm, and 1.0 × 10^−2^ S/cm, respectively. The presence of inorganic SiO_2_ and functionalized SiO_2_ enhances the membrane’s hydrophilic properties that are involved in forming H_3_O^+^ ions for transferring protons [83]. Furthermore, PVDF/sCS/fSiO_2_ composite membrane showed the highest proton conductivity due to the presence of –SO_3_H (proton conducting sites) through both proton transferring mechanisms. The –SO_3_H groups act as proton-conducting carriers through Grotthus mechanisms. Additionally, –SO_3_H groups absorb the surrounding water and enhance the WU properties, forming the proton transport channels for proton transfer. Moreover, inorganic filler –OH groups and chitosan interaction generated proton transferring channels and aided in proton transportation through vehicular mechanisms. Thus, PVDF/sCS/fSiO_2_ composite membrane exhibited the highest proton conductivity of both the Grotthus and vehicular mechanism, which has been used as a suitable PEM candidate for MFC applications. However, further modifications have been performed in the composite membrane for overcoming the limitations (specifically lower proton conductivity) and enhancing the membrane performance during MFC operation. The incorporation of structurally modified inorganic fillers (nitrogen doped, acid doped, etc.) into the polymer-based membranes acts as a proton transferring bridge that generates pathways and reduces the distance for proton hopping. Alterations such as polymer–polymer cross linking (for enhance membrane mechanical stability), functionalization (phosphorylation or sulfonation) of PVDF, etc. have been further performed to increase the MFC performances.

## 4. Conclusions

In this study, we developed the composite membrane by fabricating polyvinylidene fluoride/sulfonated chitosan/(PVDF/sCS/fSiO_2_) through a solution casting technique for proton exchange membrane in MFC applications. Initially, the sulfonation of biopolymer (CS) and functionalization of the inorganic filler (SiO_2_) were performed and confirmed by FTIR, XRD, and FESEM analysis. The chemical and structural analysis of the composite membrane revealed the homogenous incorporation and distribution of sCS and fSiO_2_. It has been identified that the composite membrane exhibited enhanced mechanical and thermal stability. After incorporating sCS and fSiO_2_, the membrane hydrophilicity has increased, which assists in the enhancement of membrane proton conductivity. It has been observed that the PVDF/sCS/fSiO_2_ composite membrane exhibited higher hydrophilic properties (WCA value of 59.2° and WU value of 31.65%). Moreover, the sulfonic acid groups, –NH_2_, –OH groups of sulfonated chitosan, and –OH functionalized hygroscopic fillers also enhanced the membrane’s hydrophilic properties. Increased hydrophilic properties of PVDF/sCS/fSiO_2_ composite membrane possessed higher IEC (0.97 meq/g) and enhanced proton conductivity (1.0 × 10^−2^ S/cm). The conductivity measurements showed that –SO_3_H groups in the composite membrane performed as proton-hopping sites for transferring protons (Grotthus mechanism). Additionally, the interaction between sCS and –OH group in fSiO_2_ created proton transferring channels (for vehicular mechanisms). Thus, PVDF fabricated with sulfonated chitosan and functionalized inorganic fillers possess potential PEM properties that could be hopefully used for enhancing the MFCs performances.

## Data Availability

Not applicable.

## Nomenclature

CS	chitosan
PEM	proton exchange membrane
sCS	sulfonated chitosan
MFC	microbial fuel cell
PVDF	polyvinylidiene fluoride
PS	1,3-propane sultone
fSiO_2_	functionalized silicone dioxide
FTIR	Fourier transform infrared spectroscopy
XRD	X-ray diffraction
λ	wavelength
FE-SEM	field emission scanning electron microscopy
TGA	thermogravimetric analysis
AFM	atomic force microscopy
TS	tensile strength
EAB	elongation at break
ASTM	American society for testing materials
WU	water uptake
SR	swelling ratio
IEC	ion-exchange capacity
σ	proton conductivity
